# Off-Target Effects of Clozapine-N-Oxide on the Chemosensory Reflex Are Masked by High Stress Levels

**DOI:** 10.3389/fphys.2019.00521

**Published:** 2019-05-22

**Authors:** Vena K. Martinez, Fatima Saldana-Morales, Jenny J. Sun, Ping Jun Zhu, Mauro Costa-Mattioli, Russell S. Ray

**Affiliations:** ^1^ Department of Pharmacology, Baylor College of Medicine, Houston, TX, United States; ^2^ Memory Brain Research Center, Baylor College of Medicine, Houston, TX, United States; ^3^ Department of Neuroscience, Baylor College of Medicine, Houston, TX, United States; ^4^ McNair Medical Institute, Houston, TX, United States

**Keywords:** clozapine-N-oxide, DREADD, chemosensory, respiration, noradrenaline

## Abstract

Respiratory chemosensory circuits are implicated in several physiological and behavioral disorders ranging from sudden infant death syndrome to panic disorder. Thus, a comprehensive map of the chemosensory network would be of significant value. To delineate chemosensory neuronal populations, we have utilized pharmacogenetic Designer Receptors Exclusively Activated by Designer Drugs (DREADD) perturbations for acute neuronal perturbations in respiratory circuit mapping. Recent studies show that the biologically inert DREADD ligand clozapine-N-oxide (CNO) is back-metabolized into the bioactive compound clozapine in rodents, emphasizing the need for CNO-only DREADD-free controls, which have been carried out in several studies. However, we show that high CNO doses used in several chemosensory circuit mapping studies nonetheless affect the chemosensory ventilatory reflexes in control mice, which is unmasked by extensive habituation. Here, unhabituated control animals showed no differences in respiratory parameters after CNO administration, whereas habituated animals receiving the commonly used dose of 10 mg/kg of CNO show a deficit in the hypercapnic (high CO_2_) chemosensory reflex, which is not present in 1 mg/kg CNO treated or saline control groups. Our findings indicate that even in appropriately controlled studies, additional masked CNO off-target effects may exist and underscore the importance of using minimal doses of activating ligand in combination with high levels of habituation.

## Introduction

The neural networks that underlie the respiratory chemosensory reflex are a primary target for understanding the etiology of several behavioral and physiological disorders. Perturbed chemosensory reflexes are hypothesized to play a role in both congenital and adult disorders including Sudden Infant Death Syndrome (SIDS), Congenital Central Hypoventilation Syndrome (CCHS), central sleep apneas, abnormal breathing in Rett Syndrome, and obesity hypoventilation syndrome (Obonai et al., 1998; Ozawa et al., 2003; Amiel et al., 2009; Ramanantsoa et al., 2011; Lavezzi et al., 2013). Under the false suffocation alarm hypothesis, inappropriate chemosensory activation or hypersensitivity is thought to play a role in subsets of patients suffering from panic disorder (Klein, 1993). Chemosensory dysfunction may also play a role in neurodegenerative diseases through sleep-disordered breathing that is associated with accelerated progression (Hakim et al., 2012; Verbraecken and McNicholas, 2013; Bahia and Pereira, 2015; Snyder et al., 2017). Thus, a better understanding of brainstem chemosensory networks will provide important clues to a number of behavioral and physiological pathologies.

Pharmacogenetic designer receptors exclusively activated by designer drugs (DREADDs) (Armbruster et al., 2007) have been employed in a number of studies to map neural populations in the respiratory chemoreflex (breathing response to elevated blood CO_2_ levels). DREADD technology in combination with intersectional genetic deployment has been utilized to silence highly targeted neuronal populations while observing respiratory function in conscious and unrestrained mice by our lab and others, avoiding many confounds from earlier circuit mapping approaches (Ray et al., 2011, 2013; Brust et al., 2014; Hennessy et al., 2017; Sun and Ray, 2017; Sun et al., 2017). These and most other studies almost always included CNO only non-DREADD expressing sibling controls that showed no chemosensory or other respiratory effects, arguing that CNO had no off-target effects on breathing in conscious and unrestrained mice despite the high dose used, 10 mg/kg. Nonetheless, it was found in several other studies that CNO and its back metabolism products [clozapine and N-desmethylclozapine (N-Des)] could have off-target effects on behavior and locomotion in a variety of assays, but respiratory output was not addressed (Guettier et al., 2009; Joober, 2010; MacLaren et al., 2016; Gomez et al., 2017; Ilg et al., 2018; Mahler and Aston-Jones, 2018; Manvich et al., 2018; Padovan-Hernandez and Knackstedt, 2018). It was also shown that CNO and its metabolites are not equivalently distributed between the circulatory system and the brain (Gomez et al., 2017).

A second, unaddressed concern arises from the stress induced by the experimental paradigm, including holding animals in a whole-body barometric plethysmography chamber, handling, intra-peritoneal injection, and rectal temperature measurements. In prior DREADD respiratory studies, naïve mice were introduced into a small chamber (140–400 mls) and allowed to acclimate 20–40 min before data acquisition (Ray et al., 2011, 2013; Brust et al., 2014; Hennessy et al., 2017; Sun and Ray, 2017; Sun et al., 2017). However, it is not clear if this amount of time is sufficient to minimize stress-induced respiratory changes that may act as an interacting factor with the effects of clozapine.

In our studies to examine the role of the noradrenergic (NA) system in respiratory control, we sought to employ the well-established *RC::P_hM4D* DREADD allele (Ray et al., 2011) to test the role of NA neurons [using *Tg(DBH-Cre)KH212Gsat (DBH-Cre) mice*] in baseline and hypercapnic respiration in unrestrained adult animals using whole-body plethysmography. Because noradrenaline and NA neurons are also known to play a central role in stress responses (Valentino and Van Bockstaele, 2008; Chen et al., 2018), we conducted a series of studies to compare extensive habituation (multiple exposures to the experimental paradigm whereby the animal learns that the experience is nonthreatening or survivable) and high vs. low (1 mg/kg) CNO doses with earlier published DREADD protocols (Ray et al., 2011, 2013; Brust et al., 2014; Hennessy et al., 2017; Sun and Ray, 2017; Sun et al., 2017). Here, we show for the first time that high systemic doses of CNO are capable of eliciting off-target effects on an autonomic respiratory function in conscious mice. We also reveal that the off-target effect of CNO on chemosensory respiratory output is effectively unmasked by extensive habituation and would not therefore be apparent in earlier CNO control studies that did not habituate animals prior to respiratory measurement, utilizing only a short acclimation period prior to data collection. Together, these results suggest that previously mapped neuronal populations may indirectly affect respiratory control through potential roles in regulating stress responses. Notably, these data align with recent reports suggesting that CNO is not biologically inert at high doses *via* metabolic conversion to clozapine (MacLaren et al., 2016; Gomez et al., 2017) and that off-target behavioral effects may manifest not only from perturbation of behavioral circuits but also from disruptions to underlying autonomic circuits and homeostasis.

## Results

### The *DBH-Cre* Driver Marks and Is Limited to TH-Expressing Noradrenergic Regions in the Brainstem That Are Inhibited by CNO Administration

**Figure 1:**
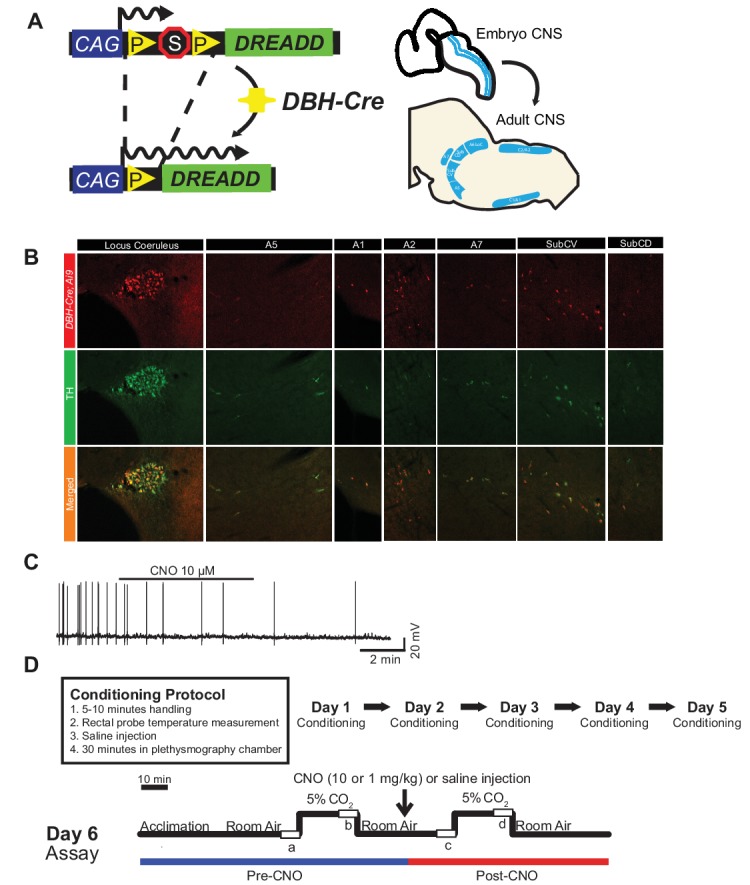
Breeding schematic and respiratory protocols. The *RC::P_hM4D* allele is combined with a *dopamine-beta-hydroxylase (DBH)-Cre* line to achieve cell-specific expression of the hM4D receptor in noradrenergic (NA) neurons **(A)**. Fluorescent expression of tdTomato in *DBH-Cre; Ai9* coronal brain sections co-expresses with tyrosine-hydroxylase (TH) in brainstem NA nuclei **(B)**. *In slice* recording of NA locus coeruleus before and after CNO application, demonstrating DREADD receptor functionality in targeted neurons **(C)**. Respiratory protocol. Habituated mice are subjected to a 5-day habituated protocol consisting of handling, rectal probe temperature measurement, saline injection, and exposure to the plethysmography chamber. On the sixth day, both habituated and unhabituated mice under a hypercapnic assay where the animal is placed into the respiratory chamber and allowed to acclimate under baseline room air conditions. The animal is then exposed to 20 min of 5% CO_2_ followed by 20 min of room air. The animal is then injected intraperitoneally with CNO or saline, followed by another 20 min of post-injection room air, 20 min of 5% CO_2_, and 20 min of room air. Open boxes delineate data collection times **(D)**.

To examine the expression and specificity of the *DBH-Cre* line, we used a single recombinase breeding scheme (Figure 1A) in which we crossed the driver to the *Ai9* line (Madisen et al., 2010), which expresses a floxed tdTomato. Staining with a tyrosine hydroxylase (TH) antibody revealed that expression of tdTomato in the brainstem was limited to TH-expressing regions, including the locus coeruleus, A5, A1, A2, A7, SubCV, and SubCD nuclei as expected (Figure 1B). To confirm that NA neurons expressing the hM4D receptor were responsive to CNO, we performed recordings on locus coeruleus (LC) neurons, where we observed an inhibition of neuron firing upon CNO bath application (*n* = 3, Figure 1C).

### CNO-hM4D Mediated Perturbation of Noradrenergic Neurons in Adult Mice

To examine the role of NA neurons under baseline and hypercapnic respiration, we employed the *RC:P_hM4D* inhibitory DREADD system crossed with the *DBH-Cre* driver. Using whole-body plethysmography (Ray et al., 2011), we measured the ventilatory responses of unrestrained adult animals under room air (21% O_2_/79% N_2_) and hypercapnic (5% CO_2_/21% O_2_/74% N_2_) conditions before and after CNO administration (Figure 1D). To address CNO dosing and potential stress induced by our experimental design, animals were subjected to one of four conditions: (1) unhabituated and injected with 10 mg/kg CNO; (2) habituated and injected with 10 mg/kg CNO; (3) unhabituated and injected with 1 mg/kg CNO; or (4) habituated and injected with 1 mg/kg CNO. Habituation consisted of a 5-day process entailing handling, rectal temperature probe, saline injection, and exposure to the plethysmography chamber each day for 30 min, while naïve animals were only exposed to a 20–45 min chamber acclimation period immediately before data collection as done in earlier studies (Ray et al., 2011, 2013; Brust et al., 2014; Hennessy et al., 2017; Sun and Ray, 2017; Sun et al., 2017). Respiratory parameters measured included respiratory rate RR, tidal volume (*V_T_*), minute ventilation (*V̇_E_*), oxygen consumption (*V̇*_O2_), minute ventilation normalized to oxygen consumption (*V̇_E_*/*V̇*_O2_), apnea frequency, sigh frequency, and coefficients of variation for interbreath interval and amplitude (periodic and volume instability). As an additional control, we also compared habituated and unhabituated wildtype animals injected with saline.

#### CNO-hM4D Perturbation of *DBH-Cre* Neurons Results in a Hypercapnic Deficit

**Figure 2:**
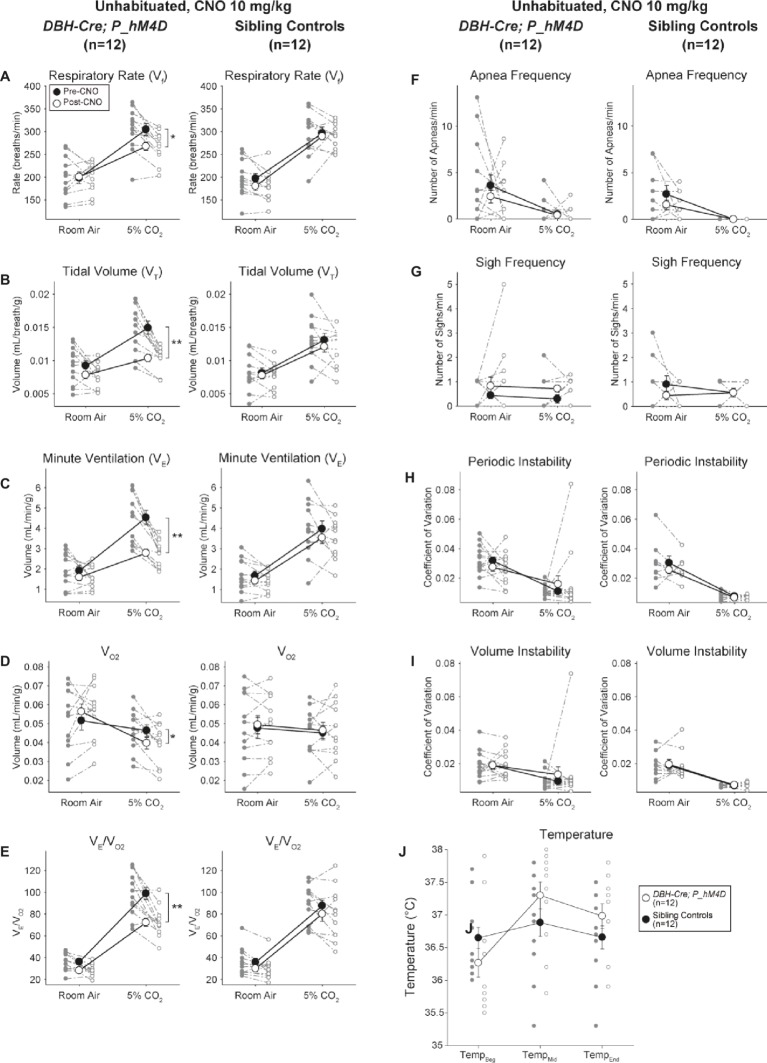
Unhabituated *DBH-Cre; RC::P_hM4D* animals administered 10 mg/kg of CNO show a hypercapnic deficit while sibling controls show no change. After CNO administration, *DBH-Cre; RC::P_hM4D* animals show no change in room air ventilation and reductions in RR **(A)**, *V_T_* **(B)**, *V̇_E_* **(C)**, *V̇*_O2_ **(D)**, and *V̇_E_/*V̇**_O2_ **(E)** under hypercapnic conditions with no changes in apnea frequency **(F)**, sigh frequency **(G)**, periodic **(H)** or volume instability **(I)**, and temperature **(J)**. Sibling controls showed no difference in all pre- and post-CNO values.

**Figure 3:**
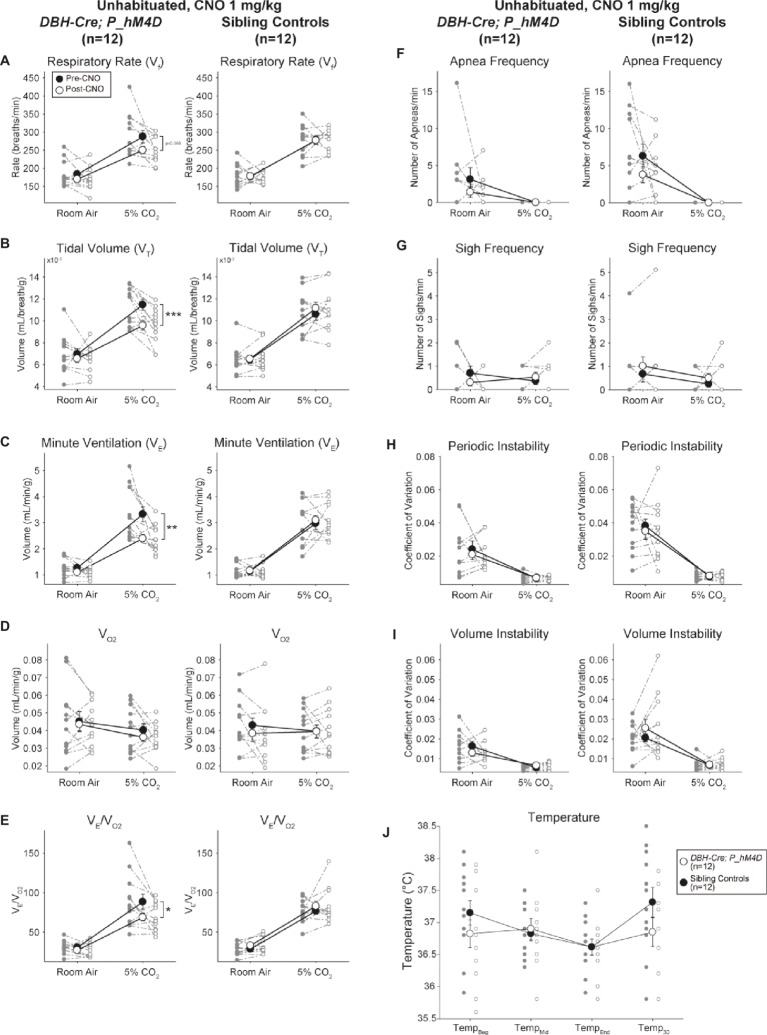
Unhabituated *DBH-Cre; RC::P_hM4D* animals administered 1 mg/kg of CNO show a hypercapnic deficit while sibling controls show no change. After CNO administration, *DBH-Cre; RC::P_hM4D* animals show no significant change in room air ventilation or RR **(A)** and showed reductions in *V_T_* **(B)**, *V̇_E_* **(C)**, and *V̇_E_/*V̇**_O2_ **(E)** under hypercapnic conditions with no changes in *V̇*_O2_ **(D)**, apnea frequency **(F)**, sigh frequency **(G)**, periodic **(H)** or volume instability **(I)**, and temperature **(J)**. Sibling controls showed no difference in all pre- and post-CNO values.

**Figure 4:**
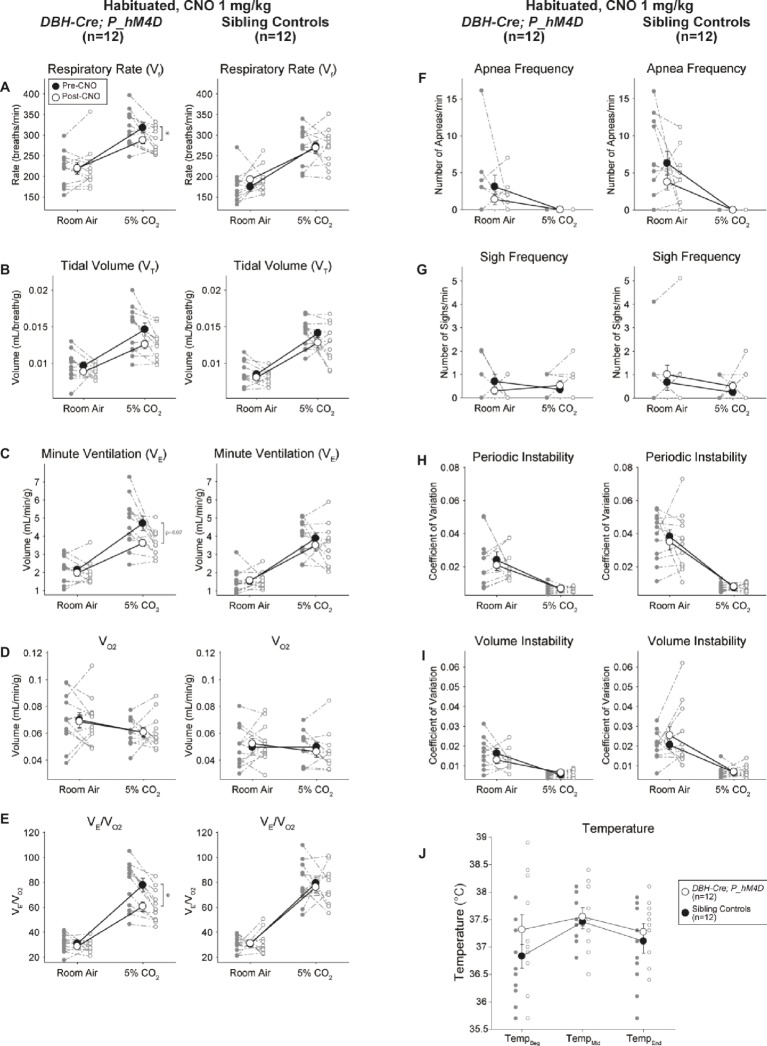
Habituated *DBH-Cre; RC::P_hM4D* animals administered 1 mg/kg of CNO show a hypercapnic deficit while sibling controls show no change. After CNO administration, *DBH-Cre; RC::P_hM4D* animals show no change in room air ventilation and a reduction in RR **(A)** and *V̇_E_/V̇*_O2_ **(E)** under hypercapnic conditions with no significant changes in *V_T_* **(B)**,*V̇_E_* **(C)**,*V̇*_O2_ **(D)**, apnea frequency **(F)**, sigh frequency **(G)**, periodic **(H)** or volume instability **(I)**, and temperature **(J)**. Sibling controls showed no difference in all pre- and post-CNO values.

Under three of the conditions, *DBH-Cre; RC::P_hM4D* animals showed a reduction in *V̇_E_* and *V̇_E_*/*V̇*_O2_ after CNO administration, while sibling controls showed no differences in pre- and post-CNO values. Unhabituated animals injected with 10 mg/kg of CNO showed a significantly reduced RR (−12.17%, *p* = 0.034) and *V_T_* (−30.87%, *p* = 0.0016), resulting in a reduction in *V̇_E_* (−38.64%, *p* = 0.0031) and a slight reduction in *V̇*_O2_ (−14.25%, *p* = 0.042) (Figure 2). The reduction in *V̇_E_* was greater than the decrease in *V̇*_O2_, resulting in an overall reduction in *V̇_E_*/*V̇*_O2_ (−26.89%, *p* = 0.0095). Unhabituated animals injected with 1 mg/kg of CNO showed a trend toward reduced RR (−12.88%, *p* = 0.066) and significantly reduced *V_T_* (−16.52%, *p* = 0.00085) and *V̇_E_* (−28.08%, *p* = 0.0070), leading to an overall reduction in *V̇_E_*/*V̇*_O2_ (−22.23%, *p* = 0.016) (Figure 3). Finally, habituated animals injected with 1 mg/kg of CNO showed a reduced RR (−10.77%, *p* = 0.074), trend toward reduced *V̇_E_* (−25.07%, *p* = 0.074), and reduced overall *V̇_E_*/*V̇*_O2_ (−23.70%, *p* = 0.024) (Figure 4). No significant changes in apnea frequency, sigh frequency, periodic or volume instability, or temperature were seen in any cohort.

#### Habituated Sibling Controls Injected With 10 mg/kg CNO Showed a Hypercapnic Ventilatory Deficit

**Figure 5:**
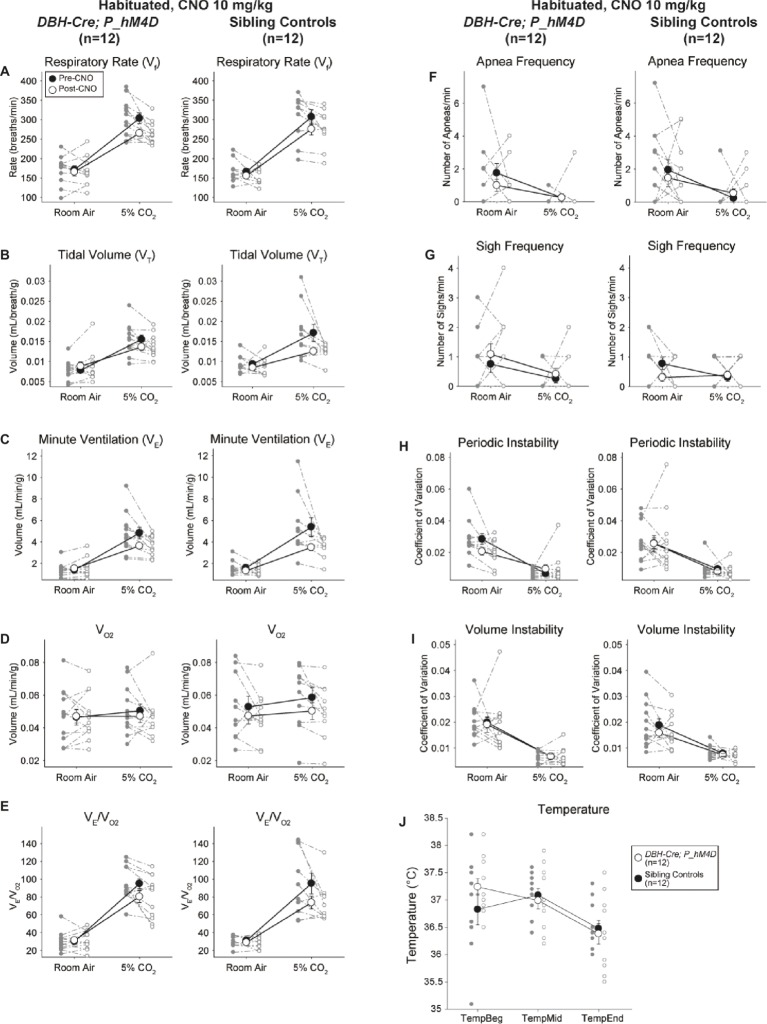
Both habituated *DBH-Cre; RC::P_hM4D* animals and sibling controls administered 10 mg/kg of CNO show a hypercapnic deficit. After CNO administration, *DBH-Cre; RC::P_hM4D* animals and sibling controls show no change in room air ventilation and a reduction in RR **(A)**, *V̇_E_* **(C)**, and *V̇_E_/V̇*_O2_ **(E)** under hypercapnic conditions with no significant changes in *V_T_* **(B)**, *V̇*_O2_ **(D)**, apnea frequency **(F)**, sigh frequency **(G)**, periodic **(H)** or volume instability **(I)**, and temperature **(J)**.

In both *DBH-Cre; RC::P_hM4D* and sibling control habituated animals injected with 10 mg/kg of CNO, we noted a significant reduction in *V̇_E_/V̇*_O2_ (*p* = 0.0235) mediated by decreases in RR (*p* = 0.00036) and *V̇_E_* (*p* = 0.037) (Figure 5). However, unlike the other cohorts, there was no difference between *DBH-Cre; RC::P_hM4D* and sibling control animals in these parameters: RR (−12.72 vs. −10.22%, *p* = 0.6268), *V̇_E_* (−24.88 vs. −23.94%, *p* = 0.4150), or *V̇_E_/V̇*_O2_ (−15.4 vs. −22.55%, *p* = 0.4643).

### Habituated and Unhabituated Wildtype Animals Injected With Saline Showed No Changes Presaline and Postsaline

**Figure 6:**
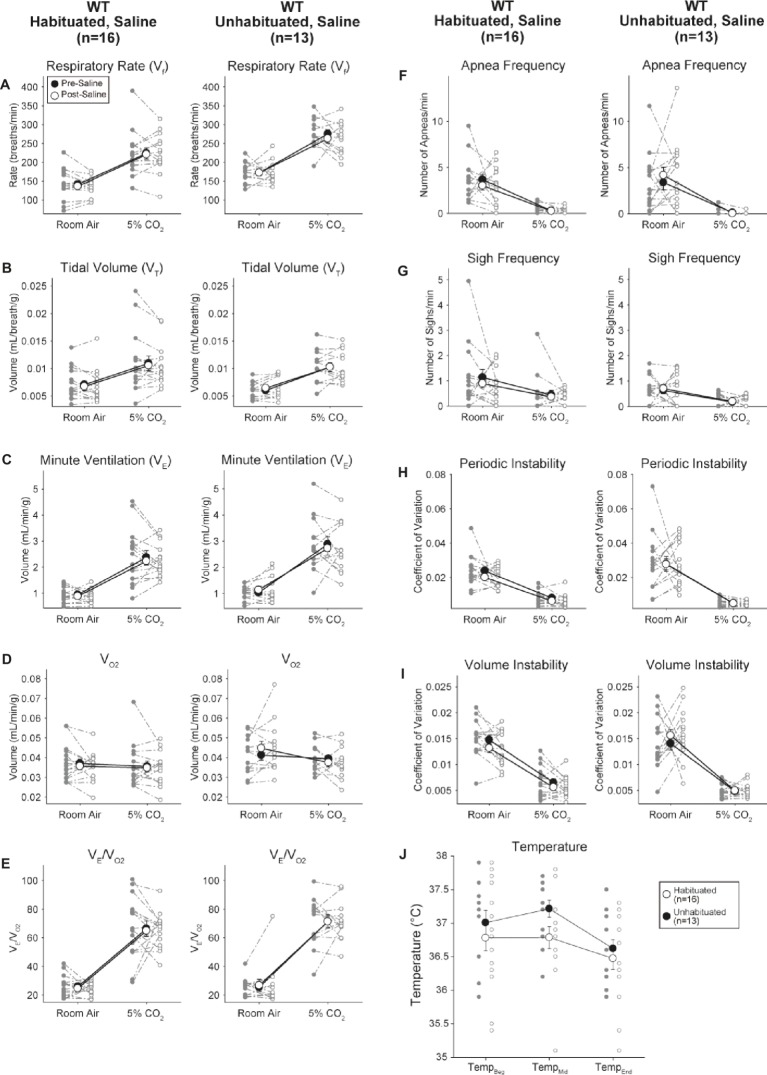
Both unhabituated and habituated wildtype animals administered saline show no difference between pre- and post-injection values. After saline administration, both unhabituated and habituated wildtype animals showed no changes under room air or hypercapnic conditions in RR **(A)**, *V_T_* **(B)**, *V̇_E_* **(C)**, *V̇*_O2_ **(D)**, *V̇_E_/V̇*_O2_ **(E)**, apnea frequency **(F)**, sigh frequency **(G)**, periodic **(H)** or volume instability **(I)**, and temperature **(J)**.

Although no phenotypes were observed in controls injected with 1 mg/kg of CNO, we addressed the possibility that the injection itself caused the phenotype observed in controls injected with 10 mg/kg of CNO by testing habituated and unhabituated wildtype animals injected with saline (Figure 6). In both habituated and unhabituated cohorts, animals did not show any difference in respiratory parameters presaline and postsaline administration. No significant changes in apnea frequency, sigh frequency, periodic or volume instability, or temperature were seen.

### Clozapine to CNO Concentration Ratios Are Higher in Brain Than in Serum

**Figure 7:**
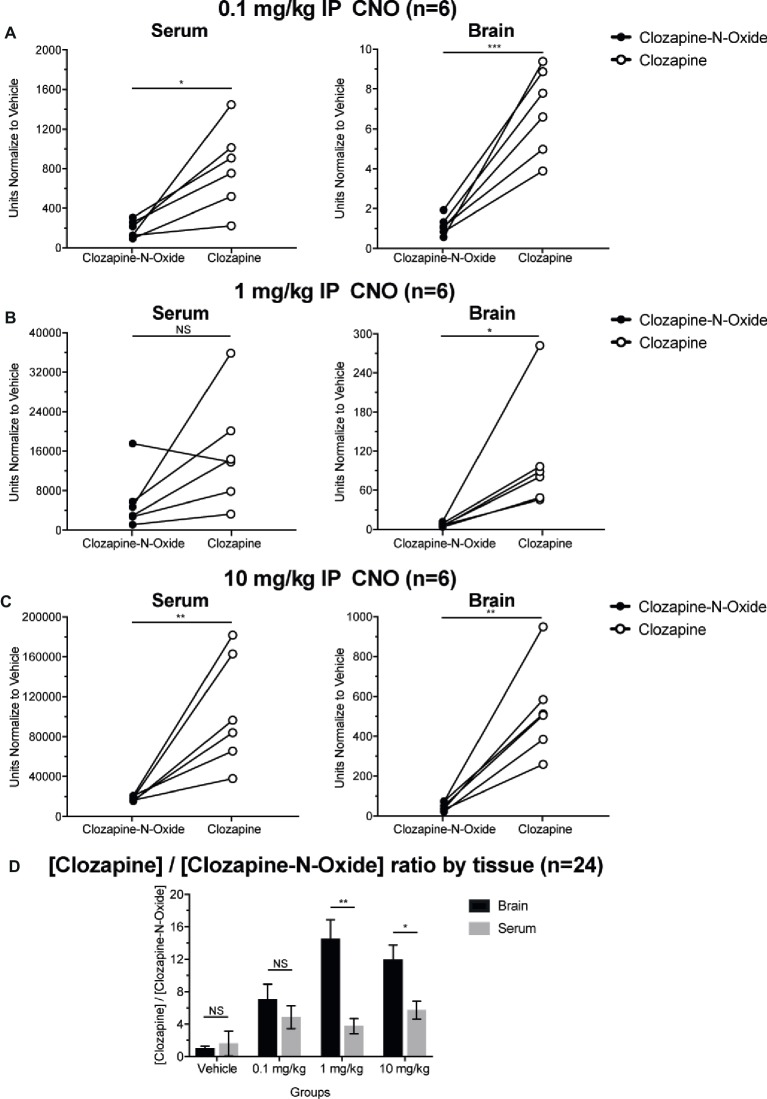
Clozapine to CNO concentrations ratios are higher in brain than in serum. Serum and brain samples were collected from wildtype mice 30 min after CNO administration at 3 different doses 0.1 mg/kg **(A)**, 1 mg/kg **(B)**, 10 mg/kg **(C)**. Individual data points represent the relative abundance of clozapine-N-oxide or clozapine obtained by LC-MS. Clozapine to CNO ratio for each dose and SEM are also shown **(D)**.

To determine bioavailability of CNO and clozapine, we measured their concentrations in serum and brain via mass spectrometry. Thirty minutes after an intraperitoneal injection of CNO in mice, CNO is found in lower relative abundance compared to its back-metabolite clozapine in the serum and brain for all doses tested, 0.1 mg/kg (serum *p* = 0.0054, brain *p* = 0.0001) (Figure 7A), 1 mg/kg (serum *p* > 0.05, brain *p* = 0.0197) (Figure 7B), and 10 mg/kg (serum *p* = 0.0036, brain *p* = 0.0005) (Figure 7C). When overall clozapine to CNO ratios were analyzed, they were always above zero and were higher in the brain than in serum (vehicle *p* > 0.5, 0.1 mg/kg *p* > 0.5, 1 mg/kg *p* = 0.0018, 10 mg/kg *p* = 0.0160) (Figure 7D).

## Discussion

The initial goal of this study was to examine the role of *DBH-Cre* neurons in respiratory physiology after acute perturbation in unrestrained and conscious adult animals. Because noradrenaline and NA neurons play a well-documented role in stress behaviors (Valentino and Van Bockstaele, 2008; Chen et al., 2018), we also sought to examine if habituation to a likely stressful physiological protocol would have an effect on the respiratory phenotypes previously observed. In the whole-body plethysmography DREADD protocol used in our lab and others, animals are handled, rectally probed for temperature, exposed to a novel environment (the plethysmography chamber), and intraperitoneally injected. Previous studies have shown that handling and habituation to injection and other “routine” procedures can modify behavioral and physiological parameters, including respiration (Misslin et al., 1982; Andrews and File, 1993; Lapin, 1995; Ryabinin et al., 1999). Other applied stressors also modify respiration under both baseline and hypercapnic ventilatory conditions (Isom and Elshowihy, 1982; Kinkead et al., 2001).

In our studies utilizing high doses of CNO, we found that hM4D-mediated inhibition of *DBH-Cre* defined NA neurons resulted in a reduced hypercapnic reflex in *V̇_E_/* *V̇*_O2_ in four defined experimental cohorts, with reduced RR, *V_T_*, and *V̇_E_*, supporting previous studies (Biancardi et al., 2008; Viemari, 2008; Gargaglioni et al., 2010). However, habituated sibling controls given a 10 mg/kg dose of CNO used in prior respiratory studies showed a ventilatory deficit under hypercapnic conditions of the same magnitude of that seen in *DBH-Cre; RC::P_hM4D* animals. No other sibling control groups showed this phenotype, including the habituated cohort that received only (1 mg/kg CNO) or saline. These results suggest that higher CNO doses (10 mg/kg) have an effect on the hypercapnic response that is unmasked after extensive habituation while presumably reducing animal stress levels, and that lower CNO doses do not have an effect on respiratory control in habituated animals. These outcomes are also in agreement with work from Korsak et al. who demonstrated that low dose CNO (2 mg/kg) does not produce off-target effects in work capacity in an exercise assay that included prior training (Korsak et al., 2018) and Fleury Curado et al. who showed no low dose CNO (1 mg/kg) specific effects on respiratory output (Fleury Curado et al., 2018).

The increased ratios of clozapine to CNO levels in serum and in the brain (Figure 7) are in concordance with recent studies that suggest that CNO is readily back metabolized to clozapine and shows greater brain permeability as compared to CNO in mice and elsewhere (Jann et al., 1994; Chang et al., 1998; Guettier et al., 2009; Gomez et al., 2017; Raper et al., 2017). However, it is not clear if the observed off-target effects are due to CNO or clozapine. As our hypercapnic measurements occurred <30 min after CNO application, it is likely that the off-target respiratory effects are mediated by clozapine. Our results (Figure 7) show high relative levels of clozapine in the brain, although CNO is not completely absent. However, Huckstepp and colleagues used direct CNO application to the ventral medulla in anesthetized rats to demonstrate that only at room air, and not under hypercapnic or hypoxic challenges, CNO application has a small effect, increasing frequency, and decreasing expiratory duration but leaving *V̇_T_* unchanged, with no clear effect observed during hypercapnia (Huckstepp et al., 2015). Given the direct application in anesthetized rats and time frame of experiments, it is likely that the small off-target effects seen were mediated by CNO and not by clozapine.

The back metabolite clozapine is a commonly used sedative and antipsychotic drug in schizophrenia with many endogenous targets, including low-high affinity antagonistic actions at D_1_, D_2_, and D_4_ dopaminergic receptors, 5-HT_2A_, 5-HT_2C_, 5-HT_3_, 5-HT_6_, and 5-HT_7_ serotonergic receptors, H_1_ histamine receptors, and α_1_ and α_2_ adrenergic receptors, among others (Fitton and Heel, 1990; Ashby and Wang, 1996). The off-target effects seen here may result from distinct or combined mechanisms and targets. Clozapine may affect respiration as a sedative. A prior study showed reduced RR and *V_T_* under 5 and 10% CO_2_ during both slow-wave-sleep and rapid-eye-movement sleep states compared to the quiet awake state in mice (Nakamura et al., 2007). Alternatively, inhibition of targeted DREADD-expressing neurons may result in an antianxiogenic or anxiolytic effect similar to our habituation protocol to reveal CNO/clozapine-mediated chemosensory off-target effects. Both explanations are supported by several studies having shown CO_2_ to play a role in innate and learned fear responses and anxiety-related behaviors (Ziemann et al., 2009; Feinstein et al., 2013; Taugher et al., 2014; Dlouhy et al., 2015; Winter et al., 2017). Thus, neurons targeted in some of these studies may indeed play a role in driving anxiogenic behavioral responses rather than physiological chemosensory reflexes as both catecholaminergic and serotonergic systems are involved in fear/anxiety behaviors and chemosensory homeostasis (Brust et al., 2014; Hennessy et al., 2017).

Conversely, the chemosensory phenotypes observed with high CNO levels may be genuine, as we were able to recapitulate the NA-mediated hypercapnic deficit at CNO doses, a magnitude of order lower in habituated mice, while control groups showed no CNO effect. However, full comparisons across earlier studies are difficult due to the lack, in some cases, of reported *V̇*_O2_, *V_T_*, RR, and *V̇* *_E_/* *V̇*_O2_ data. For example, changes to body temperature or metabolic rate may also impinge in a number of ways on respiratory and chemosensory output, and that plethysmograph chamber temperatures were vastly different in some cases (34 vs. 30°C in our studies), affecting the dynamic range of the barometric component of the waveform and thus tidal volume. Notably, we saw neither appreciable changes in respiratory waveform characteristics in any of our conditions nor acute cardio-respiratory arrest that was observed in our earlier high dose, whole rhombomere studies (Sun et al., 2017).

Our results show for the first time that CNO has an unexpected effect on the hypercapnic chemosensory reflex that is unmasked by extensive habituation. Importantly, despite high levels, a CNO off-target effect had been previously ruled out due to the lack of phenotype in sibling controls, but which we show becomes clear upon habituation. We offer an off-target characterization of CNO in the mouse model system to complement studies in rat and nonhuman primates. The results here raise the possibility that additional CNO-mediated, off-target effects on the circuits under study or to autonomic or homeostatic circuits may exist but may be masked in other controlled experiments. Importantly, these data reveal that investigators should strive to use the minimal doses of the activating ligand possible in combination with high levels of habituation, and that the proper controls must be included in chemical genetic manipulations to fully appreciate and interpret experimental data.

## Materials and Methods

### Ethical Approval

Studies were approved by Baylor College of Medicine Institutional Animal Care and Use Committee under protocol AN-6171.

### Breeding, Genetic Background, and Maintenance of Mice

We maintained colonies of all our heterozygous mouse strains by backcrossing to C57BL/6J mice and homozygous strains by sibling crosses. For histology experiments, *DBH-Cre*mice were mated with the homozygous *Ai9* mouse (Madisen et al., 2010) (JAX 007909). For plethysmography experiments, *DBH-Cre* mice were mated with homozygous *RC::P_hM4D* (Ray et al., 2011) mice to derive animals, in which all mice carried the *RC::P_hM4D* allele. Sibling animals that did not inherit the *Cre* allele were used as control animals to the *Cre* positive offspring. Rosa26 specific primers for the Ai9, *RC::P_hM4D,* and *RC::ePe* mice were 5′-GCACTTGCTCTCCCAAAGTC, 5′-GGGCGTACTTGGCATATGAT, and 5′-CTTTAAGCCTGCCCAGAAGA and yield a 495 bp band (targeted) and 330 bp band (wt). Cre-specific primers for all the rhombomere Cre drivers were 5′-ATCGCCATCTTCCAGCAGGCGCACCATTGCCC and 5′-GCATTTCTGGGGATTGCTTA and yielded a 550 bp band if positive. For LC-MS experiments, C57BL/6J mice were obtained from the Center of Comparative Medicine (CCM), Baylor College of Medicine.

### Histology

Four-to-eight-week-old *DBH-Cre; Ai9* adult mice were transcardially perfused with 0.1 M phosphate-buffered saline (PBS) and then with 4% paraformaldehyde (PFA) in PBS. Brains were dissected out and drop fixed for 2 h in 4% PFA before a PBS rinse and equilibration in 20% sucrose in PBS. Brains were then sectioned into 30 μm sections, mounted on slides, and labeled with immunofluorescent antibodies. We stained overnight with anti-tyrosine hydroxylase antibody to identify catecholaminergic neurons (1:1,000, Millipore AB152) followed by 2 h with donkey anti-rabbit Cy3 secondary (1:500, Jackson 711-165-152) in 0.1% Triton-X in PBS (PBST) with 5% donkey serum. Images were collected on a Zeiss upright epifluorescent microscope.

### Electrophysiology

Horizontal brain slices containing the locus coeruleus (300 μm) were cut with a vibratome (Leica VT 1000S, Leica Microsystems, Buffalo Grove, IL) from adult *DBH-Cre; RR2P; RC::ePe* mice (~1 month old) in 4°C artificial cerebrospinal fluid (ACSF). The slices were submerged in a chamber perfused with oxygenated ACSF (95% O_2_ and 5% CO_2_) containing in mM: 124 NaCl, 2.0 KCl, 1.3 MgSO_4_, 2.5 CaCl_2_, 1.2 KH_2_PO_4_, 25 NaHCO_3_, and 10 glucose (2–3 ml/min). Whole-cell recordings were performed at 30°C using conventional patch-clamp techniques and a MultiClamp 700B amplifier (Molecular Devices, Union City, CA). GFP-positive neurons from the locus coeruleus were visually identified and subsequently imaged by infrared differential interference contrast video on the stage of an upright microscope (Axioskope FS2, Carl Zeiss, Oberkochen, Germany). Patch pipettes (resistances 4–6 MU) were filled with (in mM): 110 K-gluconate, 10 KCI, 10 HEPES, 10 Na_2_-phosphocreatine, 2 Mg_3_-ATP, and 0.2 Na_3_-GTP; pH was adjusted to 7.2 and osmolarity to 300 mOsm. The holding potential was −70 mV. CNO was bath applied.

### Plethysmography

Plethysmography on conscious, unrestrained mice was carried out as described on 6- to 12-week-old adult animals (Ray et al., 2011). Habituated mice were subjected to a 5-day habituation protocol with each day consisting of several minutes of handling, temperature taken by rectal probe, intraperitoneal saline injection, and 30 min in the plethysmography chamber. Mice were then tested within 1 week of the last day of habituation. Unhabituated mice were not exposed to handling or the plethysmograph chamber. All mice were naïve to CNO and used only once.

On the day of testing, mice were taken from their home cage, weighed, and rectal temperature was taken. Animals were then placed into a flow-through, temperature-controlled (water jacketed at 30**°**C) plethysmography chamber and allowed to acclimate for at least 20 min in room air (21% O_2_/79% N_2_) conditions. After acclimation and measurement under room air, the chamber gas was switched to a hypercapnic mixture of 5% CO_2_/21% O_2_/74% N_2_ for 20 min. Chamber gas was then switched back to room air for 20 min. The mice were briefly removed for rectal temperature measurement and intra-peritoneal injection of CNO (National Institute of Mental Health Chemical Synthesis and Drug Supply Program) dissolved in saline (1 or 0.1 mg/ml) for an effective concentration of 10 or 1 mg/kg, respectively. The animal was returned to the chamber for another 20 min of room air, 20 min of hypercapnia, and 20 min of room air. The animal was then removed from the chamber, and rectal temperature was taken immediately after the termination of the experiment.

### Liquid Chromatography-Mass Spectrometry

Twenty-four wildtype mice, evenly divided by sex, were weighed and treated with 10 mg/kg CNO, 1 mg/kg CNO, 0.1 mg/kg CNO, or vehicle. Thirty minutes after injection, blood samples were collected via cardiac puncture and placed in BD Microtainers. Samples were centrifuged at 4°C at 13,500 rpm in bench top centrifuge, and supernatants were collected. Serum and brains were kept at −20°C until extraction.

HPLC grade solvents water, acetonitrile chloroform, and methanol and mass-spectrometry-grade formic acid were obtained from Sigma-Aldrich (St. Louis, MO). Calibration solution containing multiple calibrants in a solution of acetonitrile, trifluoracetic acid, and water was purchased from Agilent Technologies (Santa Cruz, CA). Metabolites and internal standards, including N-acetyl aspartic acid-d3, tryptophan-15N2, glutamic acid-d5, thymine-d4, gibberellic acid, trans-zeatin, jasmonic acid, 15N anthranilic acid, and testosterone-d3 were purchased from Sigma-Aldrich (St. Louis, MO). Microtainer R SST TM was obtained from Becton Dickinson (Franklin Lakes, NJ).

Extraction consisted of 750 μl of ice-cold methanol:water (4:1) containing 20 μl spiked internal standards that was added to each brain sample (50 mg) and quality controls and then was homogenized for 1 min each. Then, 750 μl of 100% acetonitrile containing 20 μl spiked internal standards were added to wash sample (100 μl) and quality controls and then sonicated for 5 min. All samples were centrifuged at 5,000 rpm for 10 min at 4°C. The resultant supernatant was collected, and 20 μl were injected into LC-MS.

All samples were analyzed using 6,490 triple quadrupole mass spectrometer (Agilent Technologies, Santa Clara, CA) coupled to HPLC system (Agilent Technologies, Santa Clara, CA) by multiple reaction monitoring (MRM). Approximately 8–11 data points were acquired per detected metabolite. Metabolites detected were clozapine, CNO, and norclozapine (N-desmethyl clozapine). ESI positive mode was used in method. The HPLC column was ACQUITYUPLC C18 column (100 Å, 1.8 μm, and 2.1 mm × 100 mm. Milford, MA, USA) with a flow rate of 0.5 ml/min.

### Data Collection and Analysis

#### Plethysmography

Plethysmography pressure changes were measured using a Validyne DP45 differential pressure transducer and reference chamber and CD15 carrier demodulator and recorded with LabChartPro in real time. Waveforms were analyzed offline using LabChartPro and custom written MATLAB code to determine respiratory rate (RR), tidal volume (*V_T_*) (Ray et al., 2011), minute ventilation (*V̇_E_*), and pattern analysis. Respiratory waveforms were collected offline during periods when the animal was at rest, and readings were free from movement artifacts. A minimum of 1 min cumulative data compiled from traces at least 10 s long from the last 10 min of a given experimental condition were analyzed. O_2_ consumption was determined by comparing the gas composition between calibration in an empty chamber and live breathing using an AEI oxygen sensor and analyzer. Chamber temperature was constantly monitored using a ThermoWorks MicroThermo 2 and probe and was recorded with LabChartPro in real time.

Poincaré measurements and sigh and apnea frequency were determined using 1 min of movement-free traces from each breathing condition. Sighs were defined as a breath with amplitude of at least twice as large as the average breath. Apneas were defined as an interbreath interval (IBI) at least twice as large as the average IBI. The coefficient of variation (CV) of the IBI and amplitude was also calculated from the same 1-min trace compilation of each breathing condition (standard error IBI or amplitude/mean IBI or amplitude).

### Statistics

#### Plethysmography

Results (RR, *V_T_,* *V̇_E_,* *V̇*_O2_, *V̇_E_/* *V̇*_O2_, number of apneas and sighs, and CVs of IBI and amplitude) for room air and hypercapnic data were compared between *DBH-Cre; RC::P_hM4D* cohorts and sibling controls using a linear mixed-effects regression model with animal type (experimental vs. control) and CNO administration (pre vs. post injection) as fixed effects and animal ID as a random effect. Temperature data were compared using a linear mixed-effects regression model with animal type (experimental vs. control) as a fixed effect. A *p* < 0.05 was used to indicate statistical significance, and individual data points, mean, and standard error of the mean are shown on all charts.

#### Liquid Chromatography-Mass Spectrometry

The obtained area under the peak for each sample was normalized by the internal control and then to vehicle before statistical analysis was performed. Unpaired *t* test was used to compare the relative abundance of clozapine and CNO in each tissue by concentration group.

## Ethics Statement

All experiments were approved by the Institutional Animal Care and Use Committee of Baylor College of Medicine. The experiments conformed to national standards for the care and use of experimental animals set by the Association for Assessment and Accreditation of Laboratory Animal Care.

## Author Contributions

JS, FS-M, MC-M and RR conceived and designed the experiments. JS, FS-M, and PZ performed the experiments and contributed to analyzing the data. JS, FS-M, VM, MC-M and RR wrote the paper.

### Conflict of Interest Statement

The authors declare that the research was conducted in the absence of any commercial or financial relationships that could be construed as a potential conflict of interest.
